# An Innovative Telemedical Network to Improve Infectious Disease Management in Critically Ill Patients and Outpatients (TELnet@NRW): Stepped-Wedge Cluster Randomized Controlled Trial

**DOI:** 10.2196/34098

**Published:** 2022-03-02

**Authors:** Gernot Marx, Wolfgang Greiner, Christian Juhra, Svenja Elkenkamp, Daniel Gensorowsky, Sebastian Lemmen, Jan Englbrecht, Sandra Dohmen, Antje Gottschalk, Miriam Haverkamp, Annette Hempen, Christian Flügel-Bleienheuft, Daniela Bause, Henna Schulze-Steinen, Susanne Rademacher, Jennifer Kistermann, Stefan Hoch, Hans-Juergen Beckmann, Christian Lanckohr, Volker Lowitsch, Arne Peine, Fabian Juzek-Kuepper, Carina Benstoem, Kathrin Sperling, Robert Deisz

**Affiliations:** 1 Department of Intensive Care Medicine and Intermediate Care, Medical Faculty RWTH Aachen Aachen Germany; 2 School of Public Health, Department of Health Economics and Health Care Management, Bielefeld University Bielefeld Germany; 3 University Hospital Muenster Muenster Germany; 4 Division of Infection Control and Infectious Diseases, Medical Faculty RWTH Aachen Aachen Germany; 5 Physician Network, Medizin und Mehr eG (MuM) Buende Germany; 6 Physician Network, Gesundheitsnetz Köln-Süd (GKS) e.V. Cologne Germany; 7 Healthcare IT Solutions GmbH Aachen Germany

**Keywords:** telemedicine, infectious disease medicine, sepsis, evidence-based medicine, eHealth

## Abstract

**Background:**

Evidence-based infectious disease and intensive care management is more relevant than ever. Medical expertise in the two disciplines is often geographically limited to university institutions. In addition, the interconnection between inpatient and outpatient care is often insufficient (eg, no shared electronic health record and no digital transfer of patient findings).

**Objective:**

This study aims to establish and evaluate a telemedical inpatient-outpatient network based on expert teleconsultations to increase treatment quality in intensive care medicine and infectious diseases.

**Methods:**

We performed a multicenter, stepped-wedge cluster randomized trial (February 2017 to January 2020) to establish a telemedicine inpatient-outpatient network among university hospitals, hospitals, and outpatient physicians in North Rhine-Westphalia, Germany. Patients aged ≥18 years in the intensive care unit or consulting with a physician in the outpatient setting were eligible. We provided expert knowledge from intensivists and infectious disease specialists through advanced training courses and expert teleconsultations with 24/7/365 availability on demand respectively once per week to enhance treatment quality. The primary outcome was adherence to the 10 *Choosing Wisely* recommendations for infectious disease management. Guideline adherence was analyzed using binary logistic regression models.

**Results:**

Overall, 159,424 patients (10,585 inpatients and 148,839 outpatients) from 17 hospitals and 103 outpatient physicians were included. There was a significant increase in guideline adherence in the management of *Staphylococcus aureus* infections (odds ratio [OR] 4.00, 95% CI 1.83-9.20; *P*<.001) and in sepsis management in critically ill patients (OR 6.82, 95% CI 1.27-56.61; *P*=.04). There was a statistically nonsignificant decrease in sepsis-related mortality from 29% (19/66) in the control group to 23.8% (50/210) in the intervention group. Furthermore, the extension of treatment with prophylactic antibiotics after surgery was significantly less likely (OR 9.37, 95% CI 1.52-111.47; *P*=.04). Patients treated by outpatient physicians, who were regularly participating in expert teleconsultations, were also more likely to be treated according to guideline recommendations regarding antibiotic therapy for uncomplicated upper respiratory tract infections (OR 1.34, 95% CI 1.16-1.56; *P*<.001) and asymptomatic bacteriuria (OR 9.31, 95% CI 3.79-25.94; *P*<.001). For the other recommendations, we found no significant effects, or we had too few observations to generate models. The key limitations of our study include selection effects due to the applied on-site triage of patients as well as the limited possibilities to control for secular effects.

**Conclusions:**

Telemedicine facilitates a direct round-the-clock interaction over broad distances between intensivists or infectious disease experts and physicians who care for patients in hospitals without ready access to these experts. Expert teleconsultations increase guideline adherence and treatment quality in infectious disease and intensive care management, creating added value for critically ill patients.

**Trial Registration:**

ClinicalTrials.gov NCT03137589; https://clinicaltrials.gov/ct2/show/NCT03137589

## Introduction

### Background

Worldwide, health workforce shortages are a pressing concern. By 2030, it is estimated that there will be a shortage of 9.9 million physicians and nurses worldwide [1,2]. In addition, the proportion of older people in Europe will exceed 30% by 2050 [3,4]. Hence, health care systems must become more flexible and efficient, for example, through accelerated digitization. Infectious disease management, especially the management of sepsis, is one area in which the potential for digitization to reduce the global burden of disease is particularly strong.

Increasing antimicrobial resistance (AMR) poses a growing threat to patients. Important underlying drivers are the overuse and misuse of antibiotics. Every year, approximately 700,000 patients worldwide die from infections that are treatable with antibiotics [5]. Global pandemics such as COVID-19 are likely to promote the overuse of antimicrobials, thereby facilitating the further development of AMR. AMR could cost US $100 trillion between now and 2050, with the annual mortality rate reaching 10 million over this period [5].

In 2017, there were approximately 48.9 million cases of sepsis worldwide and 11 million sepsis-related deaths (19.7% of all deaths globally) [6]. Sepsis is the most common cause of morbidity and mortality in intensive care units (ICUs) around the world. Although the sepsis-related mortality rate is continuously decreasing, it is still remarkably high (30%-50%) [7-9]. The associated costs are US $24 billion annually in the United States alone [6].

Numerous studies have shown that adherence to clinical practice guidelines for antibiotic therapy and sepsis management is associated with improved patient outcomes [10-15]. Alarmingly, compliance with these guidelines is low [10,16-18]. International educational health care campaigns, such as the *Choosing Wisely* initiative, have responded to this global challenge by increasing professional awareness of evidence-based medicine [19]. In general, the *Choosing Wisely* recommendations promote essential practices and the avoidance of unnecessary diagnostic, preventive, and therapeutic procedures [15,19].

Telemedicine has the potential to support these efforts. It facilitates direct, round-the-clock interactions between physicians who care for patients in hospitals with limited subspecialist staff and intensivists or infectious disease specialists located far away. Observational studies have demonstrated that expert teleconsultations can reduce sepsis-related mortality by approximately 25%, with a simultaneous increase in guideline adherence [12,20]. Despite decades of intensive care research worldwide, no drug or other therapeutic measure has achieved a comparable reduction.

### Objective

The aim of TELnet@NRW is to establish and evaluate a telemedical inpatient-outpatient network (24/7/365) to improve the application of evidence-based medicine in infectious disease management, especially the management of sepsis. We hypothesized that the establishment of a digital network based on expert teleconsultations increases treatment quality in inpatient and outpatient care for these 2 subspecialties.

## Methods

### Trial Design and Ethics Approval

TELnet@NRW was a multicenter, stepped-wedge cluster randomized trial conducted at 2 university hospitals (Aachen and Muenster), 17 hospitals and 103 outpatient physicians’ offices associated with 2 physician networks. The protocol is publicly available [21], and the trial was prospectively registered at ClinicalTrials.gov (NCT03137589). The Ethics Committee of the Medical Faculty of the RWTH Aachen approved the study (EK 068/17). The study was funded by the Innovation Fund of the Federal Joint Committee (February 2017 to January 2020, funding code 01 NVF16010). Independent researchers from the Department of Health Economics and Health Care Management at Bielefeld University conceptualized and performed the analyses. The reporting of this trial is in line with the CONSORT-EHEALTH (Consolidated Standards of Reporting Trials of Electronic and Mobile Health Applications and Online Telehealth) checklist [22].

### Inpatient and Outpatient Participants

Inpatients, aged ≥18 years, who had *Staphylococcus aureus* bacteremia or required intensive care treatment and who provided written informed consent were eligible for inclusion.

Outpatients, aged ≥18 years, with a possible infectious presentation based on the International Classification of Primary Care, who provided written informed consent were eligible for enrollment. Measles vaccination rates in children were also evaluated.

Because of the complexity and diversity of possible diagnoses and the extremely high total number of patients during the entire study period, triage for study enrollment was carried out by the attending physician on-site.

We excluded minors and patients who did not formally consent to participate in the study. In addition, patients who were in a dependent or employment relationship with the sponsor or one of the investigators or the principal investigator and patients who lived in an institution as mandated by a legal or administrative order were excluded from the study.

### Technical Requirements

Initially, the standardized technical requirements at each participating site were established in line with the relevant guideline recommendations [23]. This also included setting up a wireless local area network in hospitals and medical practices. A secure, privacy-compliant infrastructure was used for communication, including 2 high-encryption audio-video conference systems and the certified data exchange platforms *FallAkte Plus* (Healthcare IT-Solutions) and *ELVI* (CompuGroup Medical), which complied with the General Data Protection Regulation (EU Regulation 2016/679). Overall, our data protection measures were externally reviewed by independent data protection experts and continuously monitored throughout the study. The video conferencing infrastructure met high requirements in terms of quality, data security and portability. This infrastructure operated in a high-security, closed network (hardware virtual private network) or on dedicated lines.

### Interventions: Expert Teleconsultations Plus Advanced Training Courses

The innovative telemedical network TELnet@NRW involved outpatient-inpatient cooperation. Separate facilities (hospitals of different levels of care and outpatient physicians’ offices) established a new digital health care structure for North Rhine-Westphalia, Germany. TELnet@NRW provided expert knowledge from 2 university hospitals to participating hospitals and outpatient physicians through expert teleconsultations (24/7/365 availability). Expert teleconsultations were provided on request after the initial triage was carried out by the attending physician on-site. Consultants for intensive care medicine participated in key care processes 24/7, whereas infectious disease specialists were available once weekly and on demand, including participation in rounds, additional expert teleconsultations, emergency consultations, and audits of clinical patient data. Before implementation of the expert teleconsultations, participants received advanced training courses on guideline-compliant treatment.

### Study Schedule and Data Collection

All clusters went through three different study phases: During the preintervention phase, pseudonymized patient data from routine care was documented (details are provided in Multimedia Appendices 1 and 2, with the preintervention phase shown in red). During the subsequent transition phase (shown in white), the required hardware infrastructure was set up at the different study sites. Then, participants received on-site training according to their cluster schedule. To familiarize participants with the new processes, expert teleconsultations were already provided during the transition phase. Data on the effects of the intervention were collected during the following intervention phase (shown in blue). Primary data were generated using standardized case reporting forms. For the analyses of influenza and measles vaccination rates, we used routine outpatient claims data from the Association of Statutory Health Insurance Physicians (AHIP).

### Outcomes

The primary outcome measure was adherence to the 10 *Choosing Wisely* recommendations for infectious diseases provided by the German Society for Infectious Diseases, which are applicable to both inpatient and outpatient care; these contain 5 *Dos* and *Don’ts* for infectious disease management (for each definition, please refer to Multimedia Appendix 3) [15]. Notably, the first 2 *Dos* address important quality indicators in intensive care medicine as they are associated with lower mortality [12,20,24,25]. For improved overview, Textbox 1 shows the 10 *Choosing Wisely* recommendations sorted by their applicability to the inpatient and outpatient sector.

Secondary outcome measures for the inpatient sector were rate of sepsis therapy in compliance with guidelines (in compliance with the Surviving Sepsis Campaign guidelines for the management of severe sepsis and septic shock, defined as adherence to the 3- and 6-hour sepsis bundles [9]); rate of acute respiratory distress syndrome (ARDS) therapy in compliance with guidelines (measured against the evident ventilation targets, ventilation with low ventilation volumes, and low peak pressures; with controlled ventilation; breath volume of 6 mL/kg calculated ideal body weight; positive end-expiratory pressure setting in proportion with the necessary FiO_2_; and plateau pressure <30 cm H_2_O [26]); and ICU and sepsis-related mortality, hospital mortality, ICU length of stay (LOS) and hospital LOS, rate of patients with dialysis at discharge from the ICU, and rate of transfer transport (defined as rate of patients discharged to another hospital).

Secondary outcome measures for the inpatient sector were health-related quality of life (measured using the 36-Item Short Form Survey version 2.0 questionnaire).

In our protocol, the 3 process variables *rate of sepsis diagnosis*, *rate of ARDS diagnosis*, and *rate of undiagnosed sepsis* were listed as part of the secondary outcomes. We corrected this in the report. All process variables are now reported as such.

**Primary outcome sorted by sector.**

**Primary outcome inpatient sector: adherence to the following *Choosing Wisely* recommendations**

- “*Staphylococcus aureus* bloodstream infection imperatively needs efficacious antimicrobial treatment and identification and elimination of the source of infection” [P1]
- “In critically ill patients with signs of infection, early appropriate antibiotic therapy is crucial after obtaining cultures, and treatment should be regularly re-evaluated” [P2]
- “Prescribe oral forms of highly bioavailable antimicrobial agents to patients who can reliably receive and absorb medications via the enteral route” [P5]
- “Do not treat asymptomatic bacteriuria with antibiotics” [N2]
- “Do not treat Candida recovered from respiratory or gastrointestinal tract specimens” [N3]
- “Do not extend the administration of prophylactic antibiotics after surgery (after the patient has left the operating room)” [N4]
- “Do not treat elevated C‐reactive protein or procalcitonin levels in serum with antibiotics in patients not presenting signs or symptoms of infection” [N5]

**Primary outcome outpatient sector: adherence to the following *Choosing Wisely* recommendations**

- “Annual influenza vaccination should be given to individuals aged >60 years, patients with specific comorbidities, and people (eg, health care workers) who may infect vulnerable persons” [P3]
- “All children should receive the measles vaccine, and adults born after 1970 without prior documented vaccination against measles should get at least one dose of the vaccine” [P4]
- “Avoid prescribing antibiotics for uncomplicated upper respiratory tract infections including bronchitis” [N1]
- “Do not treat asymptomatic bacteriuria with antibiotics” [N2]

### Randomization and Masking

Participating hospitals and outpatient physician offices were randomly assigned to 4 clusters of 4 to 5 hospitals and 4 clusters of 23 to 28 outpatient physicians’ offices (Multimedia Appendices 1 and 2). Participants were randomly assigned to one of the clusters with different start dates for the intervention phase by an independent statistician using a computer-generated random allocation sequence. It was not possible to mask the health care staff or patients, as they were involved in the delivery of the intervention.

### Statistical Methods

Primary outcomes were evaluated using binary logistic regression models (primary data) and zero-or-one inflated beta regression models (AHIP data).

In the analyses of the inpatient data, we controlled for the treating hospital, patient age, and the Sequential Organ Failure Assessment (SOFA) score at enrollment in the study [27]. SOFA scores were calculated based on the data in routine clinical patient records. Missing baseline values for the different SOFA subscores were imputed, if available, by measurements within the first 3 days after enrollment (next observation carried backward) [28]. Only patients with complete SOFA scores (ie, measurements for all 6 subscores) at baseline were included in the analyses.

To differentiate the training effect and the external effect of expert teleconsultations from the direct counseling effect, the intervention group was separated into patients with and without expert teleconsultations, as not all patients who received interventions were treated with expert teleconsultations. Models for ICU and sepsis-related mortality and sepsis bundle compliance were specified in the same manner. Effects on the LOS were estimated using linear, gamma, and log-linear regression models with the same control variables.

In the evaluation of the primary outpatient data, we controlled for the treating outpatient physician and patient age. Three different models were estimated for each outcome:

- Model 1 contained only the group variable (group) and the control variables.
- In addition, model 2 contained a count variable (n) that recorded the number of expert teleconsultations the outpatient physician had already used before the visit with the respective patient.
- Finally, model 3 contained a quadratic term of the count variable (n2) to map possible learning curves among outpatient physicians in the sense of a decreasing marginal utility of the expert teleconsultations.

In the AHIP data, influenza and measles vaccination rates were documented quarterly at the practitioner level. To isolate the effect of the intervention on the vaccination rates, we controlled for the treating practitioner, the number of patients during the quarter and seasonal or quarterly effects.

Baseline group differences were tested using odds ratios (ORs) for binary variables or 2-tailed *t* tests for metric variables. Data cleaning and statistical analyses were performed in *R* (R Foundation for Statistical Computing; version 3.6.3) using the functions *glm* for logistic models and *gamlss* for beta regression models. CIs for regression estimates were computed using the confint command from the *stats* package, which calculated the CIs based on profile likelihood estimation. All analyses were based on a significance level of α=.05. The model structures are detailed in the Multimedia Appendix 4.

## Results

### Overview

**Figure 1:**
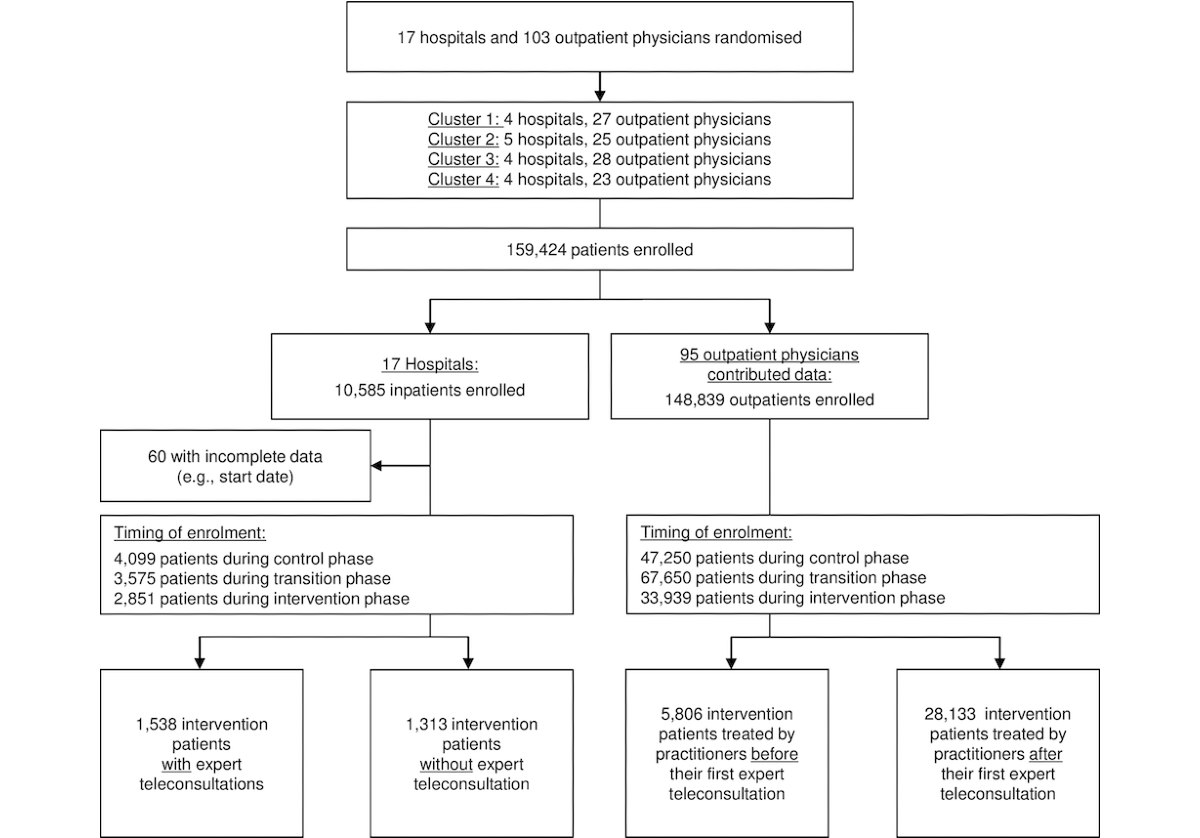
Study flow diagram.

A total of 17 hospitals and 103 outpatient physicians underwent randomization. The participating hospitals had between 101 and 449 beds; participating ICUs had on average 10 ICU beds (range 5-14) and were mixed medical-surgical ICUs staffed with anesthesiologists and internists. Hospitals were in both urban and rural areas. Of the 17 hospitals, 8 (47%) served populations <50,000; 7 (41%) served populations from 50,000 to 100,000; and 2 (12%) served populations >250,000. Most outpatient physicians were in urban areas with a high outpatient physician density per capita. Among the participating doctors, multiple specialties were represented, and the majority were general practitioners, internists, ophthalmologists, or gynecologists. Between May 3, 2017, and September 30, 2019, we enrolled patients (n=159,424) who required infectious disease or intensive care treatment in our study (Figure 1). Overall, we provided 8505 inpatient expert teleconsultations. For outpatients, the average teleconsultation rate was 1.33% (1980/148,839). In the following, we report first the results of the inpatient sector and then the results of the outpatient sector.

### Inpatient Study Enrollment

After initial triage by the attending physicians, we enrolled 10,585 inpatients (Multimedia Appendix 5). The baseline characteristics of the inpatients are detailed in Table 1. A total of 0.57% (60/10,585) of the patients with incomplete data were excluded from the analysis, and we provide details on the distribution of missing values before and after imputation (Multimedia Appendix 6). For the recommendation *Staphylococcus aureus* *bloodstream infection imperatively needs efficacious antimicrobial treatment and identification and elimination of the source of infection*, the SOFA score was not included in the statistical model, as we mainly observed non-ICU patients for this outcome, for whom the relevant parameters to calculate the score are not routinely recorded.

Compared with those in the control group, inpatients in the intervention group were older (mean age 69.25 years in the control group vs 72.14 years in the intervention group) and had higher SOFA scores at baseline (mean SOFA 3.58 in the control group vs 4.12 in the intervention group). The higher morbidity in the intervention group also manifested itself in a higher sepsis rate (5% vs 9%) and a higher ARDS rate (13% vs 17.9%). For the outcome-specific analysis samples, we found no fundamental deviations from the characteristics of the overall sample.

**Table 1:** Inpatient characteristics.

Inpatient characteristics		Control group	Transition group	Intervention group	Intervention group versus control group	
					Difference (95% CI)	*P* value
Patients, mean (SD)		4099	3575	2851	N/A^a^	N/A
Age (years), mean (SD)		69.25	70.34	72.14	2.89 (2.182-3.591)	<.001
**Sex, n (%)**						
	Male	1920 (46.8)	1616 (45.2)	1415 (49.6)	N/A	.97
	Female	2177 (53.1)	1958 (54.8)	1430 (50.2)	N/A	.97
	Other	2 (0)	1 (0)	6 (0.2)	N/A	N/A
SOFA^b^ score at baseline, mean (SD)		3.58	3.72	4.12	0.54 (0.408-0.687)	<.001
Sepsis incidence, n (%)		206 (5)	286 (8)	256 (9)	N/A	<.001
ARDS^c^ incidence, n (%)		531 (13)	696 (19.5)	511 (17.9)	N/A	<.001

^a^N/A: not applicable.

^b^SOFA: Sequential Organ Failure Assessment.

^c^ARDS: acute respiratory distress syndrome.

### Inpatient Primary Outcomes

We found significant between-group differences in the management of *Staphylococcus aureus* bloodstream infections (*Staphylococcus aureus bloodstream infection imperatively needs efficacious antimicrobial treatment and identification and elimination of the source of infection*, P1). As expert teleconsultations were provided to all patients in this analysis, we did not divide the intervention group to estimate the effects of the intervention on this recommendation. Patients in the intervention group were significantly more likely to be treated in accordance with the recommendation (OR 4.004, 95% CI 1.828-9.202; *P*=.001; Table 2).

The direct effect of expert teleconsultations became also evident in the treatment of critically ill patients with severe sepsis and septic shock. We found significant between-group differences for the recommendation *In critically ill patients with signs of infection, early appropriate antibiotic therapy is crucial after obtaining cultures, and treatment should be regularly re-evaluated* (OR 6.822, 95% CI 1.271-56.607; *P*=.04; Table 2). However, patients in the intervention group who did not receive expert teleconsultations also received treatment that was more in line with the guideline recommendation than that received by the control group. Notably, across all included patients diagnosed with severe sepsis and septic shock, adherence to this recommendation was negatively associated with patient age (Table 2).

We found no significant intervention effects for the recommendation *Prescribe oral forms of highly bioavailable antimicrobial agents to patients who can reliably receive and absorb medications via the enteral route* (Table 2).

Regarding the recommendations *Do not treat asymptomatic bacteriuria with antibiotics* (n=24) and *Do not treat Candida recovered from respiratory or gastrointestinal tract specimens* (n=32), we had too few observations to generate logistic regression models (Table 2).

Regarding the extension of the period of treatment with prophylactic antibiotics after surgery once a patient has left the operating room, we observed a higher guideline adherence in patients in the intervention group who did not receive expert teleconsultations than in patients in the control group (OR 9.372, 95% CI 1.519-111.467; *P*=.04; Table 2). However, the estimated coefficient for the portion of the intervention group who directly received expert teleconsultations remained statistically nonsignificant.

Furthermore, no significant intervention effects could be found for the recommendation *Do not treat elevated C‐reactive protein or procalcitonin levels in serum with antibiotics in patients not presenting signs or symptoms of infection* (Table 2). It should be noted that the latter was already fulfilled in 90% (531/590) of the control cases.

**Table 2:** Regression analyses of inpatient primary outcomes.^a^

		P1^b^ (N=186)				P2^c^ (N=211)				P5^d^ (N=126)				N4^e^ (N=193)				N5^f^ (N=919)		
		Compliance, % (n/N)	OR^g^ (95% CI)	*P* value	Compliance, % (n/N)		OR (95% CI)	*P* value	Compliance, % (n/N)		OR (95% CI)	*P* value	Compliance, % (n/N)		OR (95% CI)	*P* value	Compliance, % (n/N)		OR (95% CI)	*P* value
**Control variables**																				
	SOFA^h^ score	—^i^	—	—	N/A^j^		0.973 (0.863-1.096)	.65	N/A		1.355 (1.064-1.787)	.02	N/A		1.164 (0.753-1.879)	.51	N/A		0.772 (0.608-0.975)	.03
	Age	N/A	0.973 (0.944-1.000)	.06	N/A		0.952 (0.914-0.987)	.01	N/A		0.995 (0.953-1.042)	.82	N/A		1.048 (1.008-1.093)	.02	N/A		0.993 (0.979-1.007)	.34
**Group variables**																				
	Control group	16.3 (15/92)	Ref^k^	N/A	49.2 (29/59)		Ref	N/A	21.6 (19/88)		Ref	N/A	85.3 (110/129)		Ref	N/A	90.0 (531/590)		Ref	N/A
	Intervention group	45.7 (43/94)	4.004 (1.828-9.202)	<.001	—		—	—	—		—	—	—		—	—	—		—	—
	Without teleconsultation	—	—	—	81.8 (108/132)		4.718 (2.032-11.563)	<.001	0.00 (0/14)		0.000 (0.000, 1.032)	.99	93.9 (31/33)		9.372 (1.519-111.467)	.04	84.9 (163/192)		0.990 (0.542-1.834)	.97
	With teleconsultation	—	—	—	90.0 (18/20)		6.822 (1.271-56.607)	.04	25.0 (6/24)		1.135 (0.179, 7.493)	.89	80.6 (25/31)		1.744 (0.326, 12.861)	.54	92.1 (125/137)		1.463 (0.666-3.416)	.36

^a^Each model also controlled for hospital specific effects, which are not reported individually in this table; CIs were calculated based on profile likelihood estimation.

^b^Primary outcome P1: Imperatively start antimicrobial treatment and remove the focus on *Staphylococcus aureus* bloodstream infection.

^c^Primary outcome P2: Critically ill patients with signs of infection need early appropriate antibiotic therapy.

^d^Primary outcome P5: Prefer oral formulations of highly bioavailable antimicrobials whenever possible.

^e^Primary outcome N4: Do not prolong prophylactic administration of antibiotics in patients after they have left the operating room.

^f^Primary outcome N5: Do not treat an elevated C‐reactive protein or procalcitonin level with antibiotics in patients without signs of infection.

^g^OR: odds ratio.

^h^SOFA: Sequential Organ Failure Assessment.

^i^Due to differences model specifications, the respective variables were not included in all models.

^j^N/A: not applicable.

^k^Ref: reference group.

### Inpatient Secondary Outcomes

**Figure 2:**
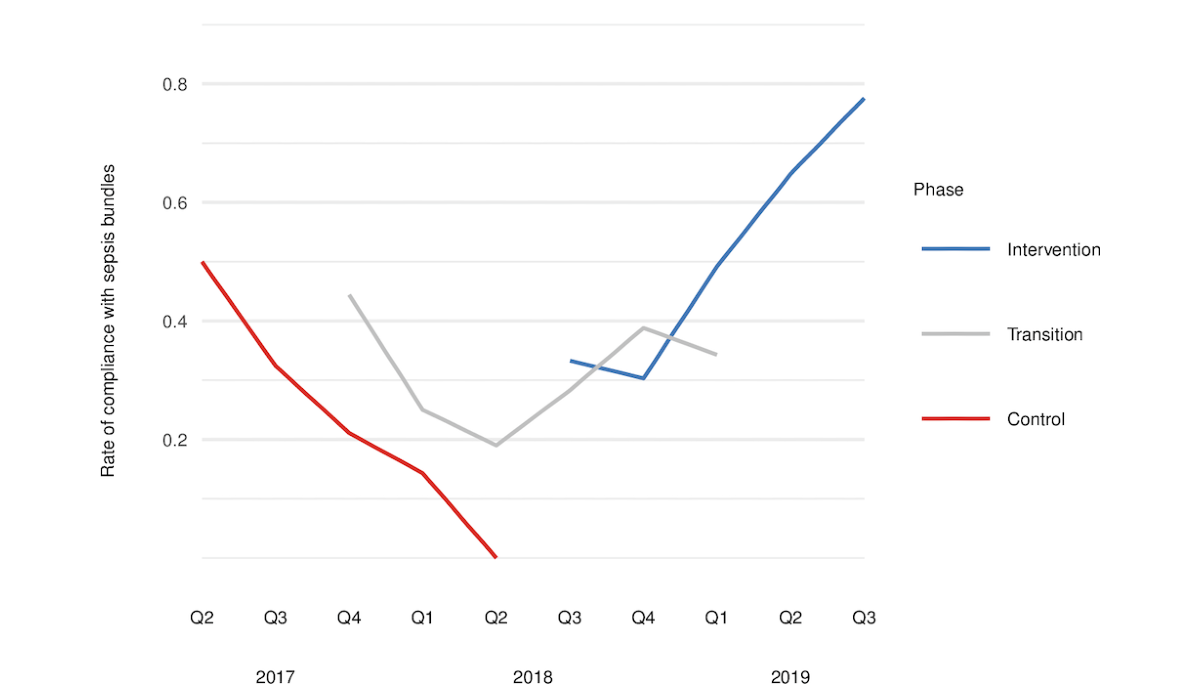
Sepsis bundle compliance over time.

Significant between-group differences were also found in the adherence to the 3- and 6-hour sepsis bundles for patients with the diagnoses of severe sepsis and septic shock (Figure 2 and Multimedia Appendix 7). Overall (0-6 hours), patients in the intervention group with expert teleconsultations outperformed the controls with regard to sepsis bundle compliance (OR 7.739, 95% CI 2.379-28.026; *P*=.001). This was mainly driven by an improvement in compliance with the 4 to 6-hour bundle. With expert teleconsultations, the odds of being treated in accordance with the guideline recommendations in the 4- to 6-hour therapy course after receiving a diagnosis of sepsis were 14.2 times higher (OR 14.245, 95% CI 3.121-85.424; *P*=.001) than in the control group. Overall and for the 3- and 6-hour sepsis bundles, we found significant improvements in compliance for patients in the intervention group who did not receive direct telemedical treatment support.

Regarding treatment quality in patients with mild ARDS, the descriptive analysis showed an increase in compliance from 7.4% (16/217) in the control group to 18.4% (9/49) in the intervention group without expert teleconsultations and 11.8% (19/161) in the group with expert teleconsultations. In the logistic regression model, a significant OR of 3.621 (95% CI 1.256-10.319; *P*=.02) was obtained for patients without expert teleconsultations. Patients with expert teleconsultations also showed a significantly increased chance of correct treatment mild ARDS (OR 2.355, 95% CI 1.023-5.516; *P*=.04). For patients with moderate or severe ARDS, we could not demonstrate these effects. A detailed description of this outcome is presented in Multimedia Appendix 8.

Regarding ICU mortality (OR 1.276, 95% CI 0.909-1.794; *P*=.16) and sepsis-related mortality (OR 0.680, 95% CI 0.230-10.874; *P*=.37), no statistically significant intervention effects were found in our model estimations; however, a reduction in the sepsis-related mortality rate of 5% was achieved (19/66, 28.8% in the control group vs 50/210, 23.8% in the intervention group). Also, no significant improvements with regard to hospital mortality were observed.

ICU LOS was significantly longer for intervention patients with (+1.971 days, 95% CI 1.858-27.708; *P*=.004) and without (+2.253 days, 95% CI 2.235-40.535; *P*=.002) expert teleconsultations than for the respective controls. Regarding hospital LOS, it is noticeable that patients in the control group were hospitalized longer (mean 16.3 days, 95% CI 15.65-16.97 days) than patients in the intervention group without expert teleconsultations (mean 14.15 days, 95% CI 12.96-15.35 days) but for shorter periods than patients with expert teleconsultations (mean 20.62 days, 95% CI 19.55-21.70 days). The linear regression model confirms this result for patients with expert teleconsultations. These patients stayed on average 4.6 days (β=4.610 days, 95% CI 3.316-5.905 days; *P*<.001) longer in hospital than control patients. For the group of patients without expert teleconsultations, there was no significant difference to the control group.

A total of 0.3% (6/1983) of the patients in the control group and 1.1% (8/739) in the intervention group were discharged from hospital on dialysis. With this very low prevalence, effects of any intervention cannot be shown.

Overall, 4.38% (86/1965) of the patients in the control group were transferred to another hospital during our study. In the intervention group, this proportion was 11.34% (143/1261). Patients with expert teleconsultations were transferred more frequently (101/857, 11.8%) than patients in the intervention group without expert teleconsultations (42/404, 10.4%). The model calculation also shows a significant intervention effect. Patients who received an expert teleconsultation had a 2.9-fold higher chance of being transferred (OR 2.903, 95% CI 2.012-4.186; *P*<.001). In patients in the intervention group without expert teleconsultations, this chance was also significantly increased compared with that in the control group (OR 2.432, 95% CI 1.570-3.721). Overall, the analyses thus show an increase in the number of transfers owing to the intervention.

### Outpatient Study Enrollment

In the outpatient sector, 148,839 patients were enrolled in our study. The intervention group differed significantly from the control group with regard to their distribution between the 2 physician networks. Baseline characteristics are detailed in Table 3, and Multimedia Appendix 9 provides details concerning study enrollment.

**Table 3:** Outpatient characteristics.

Outpatient characteristics		Control group	Transition group	Intervention group	Intervention group versus control group	
					Difference (95% CI)	*P* value
Patients, mean (SD)		47,250	67,650	33,939	N/A^a^	N/A
Age (years), mean (SD)		42.08	40.50	42.20	0.12 (−0.4538 to 0.2208)	.49
**Sex, n (%)**						
	Male	25,908 (54.8)	36,962 (54.6)	18,584 (54.8)	N/A	.83
	Female	21,342 (45.2)	30,688 (45.4)	15,355 (45.2)	N/A	.83
**Physician network, n (%)**						
	MuM^b^	31,248 (66.1)	43,412 (64.2)	21,388 (63)	N/A	<.001
	GKS^c^	16,002 (33.9)	24,238 (35.8)	12,551 (37)	N/A	<.001

^a^N/A: not applicable.

^b^MuM: Medizin und Mehr eG.

^c^GKS: Gesundheitsnetz Köln-Süd eV.

### Outpatient Primary Outcomes

Use of expert teleconsultation was associated with a significantly higher degree of adherence to the guideline recommendations for antibiotic therapy. Overall, the treatment of patients in the intervention group was significantly more compliant than that in the control group with regard to the *Choosing Wisely* recommendation for the treatment of uncomplicated upper respiratory tract infections (N1; OR 1.343, 95% CI 1.155-1.562; *P*=.001; model 1). This effect was significantly influenced by the number of expert teleconsultations conducted, as indicated by the estimated coefficient of the count variable (OR 1.007, 95% CI 1.001-1.013; *P*=.04; model 2). In addition, the estimated coefficient for the quadratic term of the count variable showed a statistically significant negative effect, which illustrates the decreasing marginal utility of the expert teleconsultations (OR 0.9998, 95% CI 0.9996-0.9999; *P*=.001; model 3). Here, we also observed an accumulation of noncompliance in the treatment of older patients (Table 4).

Our telemedical inpatient-outpatient network also achieved significant results with regard to the management of asymptomatic bacteriuria (N2). Patients in the intervention group were more likely to be treated in line with the guideline recommendations than were the controls (OR 9.312, 95% CI 3.794-25.936; *P*<.001; model 1). This effect was influenced more by the number of expert teleconsultations conducted than by the training of the treating physicians (OR 1.533, 95% CI 1.212-2.190; *P*=.004; model 2). Although not statistically significant, we also observed a trend toward a decreasing marginal utility of the expert teleconsultations with regard to this outcome (model 3). The *Choosing Wisely* recommendations also provide advice for increasing influenza (P3) and measles vaccinations (P4). For these analyses, we examined the quarterly vaccination rates at the physician level during the study period. In our basic model, we found no significant effect of expert teleconsultations on influenza vaccinations (rate ratio 1.089, 95% CI 0.911-1.302; *P*=.34). However, to better capture seasonal effects on the vaccination rates, we additionally constructed a model in which we extended the observation period and considered the transition phase as part of the intervention phase because the expert training occurred at the end of the control phase. In this model, intervention physicians had significantly higher influenza vaccination rates (rate ratio 1.204, 95% CI 1.079-1.344; *P*=.001). Regarding measles vaccination rates, we found no significant intervention effects in either model.

**Table 4:** Regression analyses of outpatient primary outcomes.^a^

			Compliance, % (n/N)		Model 1					Model 2					Model 3		
					OR^b^ (95% CI)		*P* value		OR (95% CI)			*P* value		OR (95% CI)			*P* value
**N1^c^ (N=15,714)**																	
	Age	N/A^d^		0.978 (0.975-0.980)		<.001		0.978 (0.975-0.980)			<.001		0.978 (0.975-0.980)			<.001	
	Control group	80.4 (7606/9456)		Ref^e^		N/A		Ref			N/A		Ref			N/A	
	Intervention group	90.2 (5643/6258)		1.343 (1.155-1.562)		<.001		1.198 (0.997-1.438)			.05		0.999 (0.806-1.238)			.99	
	Number of teleconsultations	—^f^		—		—		1.007 (1.001-1.013)			.03		1.032 (1.015-1.049)			<.001	
	Squared number of teleconsultations	—		—		—		—			—		0.9998 (0.9996-0.9999)			.001	
**N2^g^ (N=752)**																	
	Age	N/A		0.996 (0.983-1.010)		.55		0.999 (0.985-1.012)			.83		0.999 (0.985-1.012)			.84	
	Control group	54.5 (145/266)		Ref		N/A		Ref			N/A		Ref			N/A	
	Intervention group	75.9 (369/486)		9.312 (3.794-25.936)		<.001		0.147 (0.010-1.218)			.11		0.092 (0.002-2.639)			.16	
	Number of teleconsultations	—		—		—		1.533 (1.212-2.190)			.004		1.717 (0.819-3.174)			.08	
	Squared number of teleconsultations	—		—		—		—			—		0.994 (0.978-1.038)			.65	

^a^Each model also controlled for physician-specific effects, which are not reported individually in this table; CIs were calculated based on profile likelihood estimation.

^b^OR: odds ratio.

^c^Primary outcome N1: Avoid prescribing antibiotics for uncomplicated upper respiratory tract infections.

^d^N/A: not applicable.

^e^Ref reference group.

^f^Due to the different model specification, the respective variables were not included in all models.

^g^Primary outcome N2: Do not treat asymptomatic bacteriuria with antibiotics.

### Outpatient Secondary Outcome

We had planned to assess health-related quality of life in outpatients measured with the 36-Item Short Form Survey version 2.0 questionnaire. Completed questionnaires from a total of 540 patients were available for the initial survey time t0 (study enrollment). This corresponds to 0.4% (540/148,839) of the patients included in the outpatient study. Of the 540 patients, 72 (13.3%) were eligible for a further survey after 3 or 12 months, as the contact data required for a renewed contact were collected for them at t0. The response rates in the follow-ups for these 72 patients were 32% (n=23) for t1 and 28% (n=20) for t2. An analysis of changes over time did not appear to be appropriate based on the low response rates for t1 and t2, and no analysis was performed.

## Discussion

### Summary of Main Findings

To the best of our knowledge, TELnet@NRW is the largest telemedical cluster randomized controlled study in Europe, with more than 150,000 patients. We established a telemedical inpatient-outpatient network as a novel digital structure in the health care system, and we found that it measurably improved the quality of patient care. The consistent introduction and implementation of standardized communication using a certified electronic patient record was a feature that was essential for increasing the effectiveness of the processes involving the new digital health network. The key findings of this study suggest that expert teleconsultation is an effective tool to provide inpatient and outpatient physicians with evidence-based expertise on a large scale, thus improving guideline compliance and the quality of infectious disease and intensive care management.

Concerning the inpatient primary outcomes, our telemedical intervention had significant quality-improving effects on the management of *Staphylococcus aureus* bloodstream infections (P1), severe sepsis and septic shock (P2), and prophylactic antibiotic therapy (N4). Quality improvements for several outcomes reached not only those patients who were treated with direct expert teleconsultations but also other patients treated by the same physicians. This finding can be interpreted as a positive effect of the initial training courses and/or an indirect effect of the expert teleconsultations. It can therefore be assumed that the treating physicians also apply the knowledge acquired in teleconsultations to patients whose treatment is carried out without telemedical support.

In the outpatient sector, our telemedical intervention significantly increased guideline compliance in the management of uncomplicated upper respiratory tract infections (N1) and asymptomatic bacteriuria (N2). The chance of being treated according to the recommendations was positively associated with the number of teleconsultations already participated in by the outpatient physician before the respective patient visit. Furthermore, we found evidence for a decreasing marginal utility of these teleconsultations, which may reflect the physicians’ learning curve. To obtain a better understanding of physicians’ learning behavior and the associated practical implications, studies with longer observational periods are needed. Basic analyses of influenza and measles vaccination rates uncovered no significant intervention effects. However, when considering the initial transition phase as part of the intervention phase, influenza vaccination in the intervention group increased significantly. This could be explained either by the better statistical control of seasonal effects (as more observation quarters were included) or by the fact that the effects were larger immediately after the start of the intervention. The latter would indicate that the transition period was probably too long to capture the full potential of our intervention. Nevertheless, as this analysis deviates from the original study plan, it can only be interpreted as exploratory.

With regard to our secondary outcomes analyzed for the inpatient sector, we found that the provision of expert teleconsultations led to a higher overall sepsis bundle adherence, which was mainly driven by improvements in compliance with the 4- to 6-hour bundle. However, although the direct relationship between sepsis bundle compliance as a quality-of-care indicator and mortality is well documented in the scientific literature [10-15,20,29], we did not observe significant intervention effects on ICU mortality, sepsis-related mortality, or hospital mortality.

Although its cross-sectoral applicability was an argument for selecting compliance with the *Choosing Wisely* recommendations as the primary outcome of this study, parts of these recommendations are not applicable to both inpatient and outpatient care, especially the ICU setting. Because gastrointestinal function is very often impaired in patients in the ICU and therefore the absorption of oral medications cannot be guaranteed, most medication is administered intravenously. Likewise, obtaining tracheal secretions and microbiological tests is a rarity in the outpatient sector, as the therapeutic consequence outside of serious infections is very low. As most patients in the ICU have an indwelling urinary catheter and are sedated, the criterion *asymptomatic* cannot be evaluated in the context of bacteriuria. Notably, the compliance with abstaining from treating elevated C‐reactive protein or procalcitonin levels in the serum with antibiotics in patients without signs or symptoms of infection was already 90% (531/590) in the control phase. In summary, 40% (4/10) of *Choosing Wisely* recommendations for infectious disease management are not fully applicable to ICU care.

Within the real-world setting of our trial, it was at the discretion of the practitioners for which patients an expert teleconsultation was requested. Hence, attending physicians tended to include patients thought to be more ill during the intervention phase of the study, which is reflected by the higher SOFA scores, sepsis, and ARDS incidences for inpatients in the intervention phase compared with those in the control phase. We controlled for such selection effects by including relevant variables in our regression models, but we could not rule out the presence of unknown confounders for which there were no data available. Although our regression models were adjusted for the SOFA score, this may not have fully controlled for the differences in the baseline risks of morbidity and mortality between the study groups. It is unclear to what extent this problem has been aggravated by the frequent occurrence of missing values for the different SOFA subscores and the associated need for data imputation. It should be highlighted that TELnet@NRW was not designed or powered to detect differences in sepsis-related mortality because the primary outcome focused on quality indicators for infectious disease management (Multimedia Appendix 3). Nevertheless, in the treatment of patients with sepsis, the early detection of sepsis followed by the early initiation of therapy conforming to the recommendations in the guidelines significantly improves the clinical outcomes [8,10,20,24,30]. Hence, if expert teleconsultations continue to be part of routine health care, we expect that this will also have a positive effect on mortality in ICUs, as has been reported in similar trials in the past [12,20,24]. The equivalent is well documented in the literature regarding adherence to evidence-based management of *Staphylococcus aureus* bacteremia. Guideline adherence significantly improves clinical outcomes and reduces mortality [31-35].

As Hemming et al [36] note, control for secular effects plays a crucial role in stepped-wedge trials. However, especially the need for an appropriately long transition phase to implement the intervention at the participating sites impeded an adequate mapping of time in our statistical models. To address this shortcoming, we estimated secular effects on our inpatient primary outcomes under consideration of transition phase data in a secondary analysis. Our models incorporate time in two ways: (1) the days since the beginning of the study (ie, the beginning of the control phase) and (2) the days since the beginning of the transition phase. Results of the estimations are displayed in the Multimedia Appendix 10. For most of the primary outcomes, we observed no significant time effects (P5, N4, and N5). The estimation for P2 shows (1) a significant negative overall time effect and (2) a significant positive effect since the beginning of the implementation of the intervention. Thus, we conclude that our intervention was a major driver of the observed improvements and that our primary model without time variables may rather tend to underestimate the intervention effects. For outcome P1, however, the estimation shows significant time effects pointing in the opposite direction. It can be assumed that the negative time effect observed since the beginning of the transition phase is largely due to implementation difficulties in the participating normal wards. However, we cannot rule out that the intervention effects measured in the primary evaluations were overestimated owing to a possible time trend.

### Limitations

Overall, the ﬁndings of this study should be interpreted in the context of its limitations. The participating hospitals, ICUs, and outpatient physicians were not chosen at random; instead, the 17 sites and 103 outpatient physicians were self-selected based on their willingness to participate in a study to improve patient care. However, we chose a randomized stepped-wedge design to control for clinical characteristics, demographics, and setting to protect our findings against bias. Nevertheless, our results should be interpreted with the consideration of the possibility for selection bias due to the on-site triage of patients. We controlled for such selection effects by including relevant variables in our regression models, but we could not rule out the presence of unknown confounders for which there were no data available. We also acknowledge potential bias related to secular trends in care given the fact that the control and intervention clusters did not overlap in time. Furthermore, for some outcomes, the real effect size remained uncertain, which is reflected by the large CI. This holds true especially for sepsis bundle compliance. Nevertheless, there is sufficient certainty that the associated ORs were >1; thus, intervention effects existed.

### Conclusions

Despite the mentioned limitations, TELnet@NRW robustly demonstrated that a cross-sectoral health network, as a new digital structure in the health care system, can develop into a quality network that operates under the premise that cross-sectoral and interregional cooperation can significantly improve evidence-based care. On the basis of the technical equipment, the principles of TELnet@NRW are transferable from intensive care medicine and infectious disease management to other subspecialist medical fields that mostly rely on expert knowledge. Thus, the concept of TELnet@NRW can be adapted to other patient populations, other conditions, or other areas, in which expertise rather than equipment needs to be transported over large distances. Our results must also be interpreted in light of the most recent SARS-CoV-2 pandemic; a digital inpatient-outpatient health network is well suited to meeting pressing challenges faced by health care systems, which we will have to address in the future (eg, staff shortages in health care sectors, lack of experts in the geographic area, and aging societies). However, further research is needed with regard to the long-term patient-relevant effects of our telemedical solution and its cost-effectiveness especially in less complex cases in the outpatient setting.

## Appendix

Multimedia Appendix 1 Study schedule using a stepped-wedge design (inpatient sector).

Multimedia Appendix 2 Study schedule using a stepped-wedge design (outpatient sector).

Multimedia Appendix 3 Analysis algorithms of the 10 *Choosing Wisely* recommendations.

Multimedia Appendix 4 Model structures.

Multimedia Appendix 5 Study enrollment over time (inpatient sector).

Multimedia Appendix 6 Missing value distribution of baseline Sequential Organ Failure Assessment scores.

Multimedia Appendix 7 Regression analyses of sepsis bundle compliance.

Multimedia Appendix 8 Regression analysis of acute respiratory distress syndrome therapy compliance.

Multimedia Appendix 9 Study enrolment over time (outpatient sector).

Multimedia Appendix 10 Regression analyses of secular effects on inpatient primary outcomes.

Multimedia Appendix 11 TELnet@NRW Study Group.

Multimedia Appendix 12 CONSORT-EHEALTH Checklist (V 1.6.1).

## Abbreviations

AHIP: Association of Statutory Health Insurance Physicians
AMR: antimicrobial resistance
ARDS: acute respiratory distress syndrome
CONSORT-EHEALTH: Consolidated Standards of Reporting Trials of Electronic and Mobile Health Applications and Online Telehealth
ICU: intensive care unit
LOS: length of stay
OR: odds ratio
SOFA: Sequential Organ Failure Assessment
