# Supplementary figures and images for: An Innovative Telemedical Network to Improve Infectious Disease Management in Critically Ill Patients and Outpatients (TELnet@NRW): Stepped-Wedge Cluster Randomized Controlled Trial
Source: J Med Internet Res. 2022 Mar 2;24(3):e34098. doi: 10.2196/34098 (PMC8928042; doi:10.2196/34098)

Multimedia appendix 1, Study schedule using a stepped-wedge design (inpatient sector)


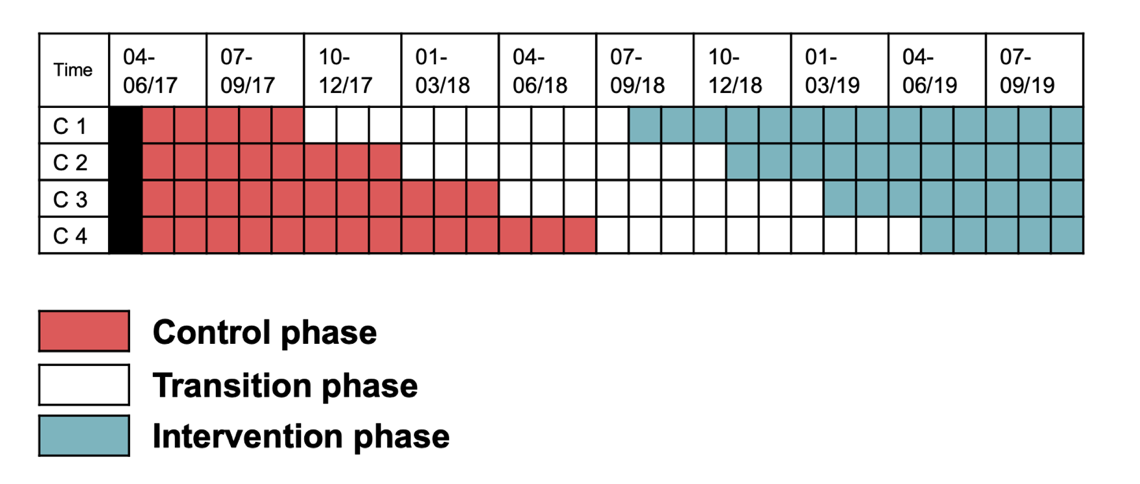

Supplement: Multimedia Appendix 1 [file jmir_v24i3e34098_app1.docx]

Multimedia appendix 2, Study schedule using a stepped-wedge design (outpatient sector)


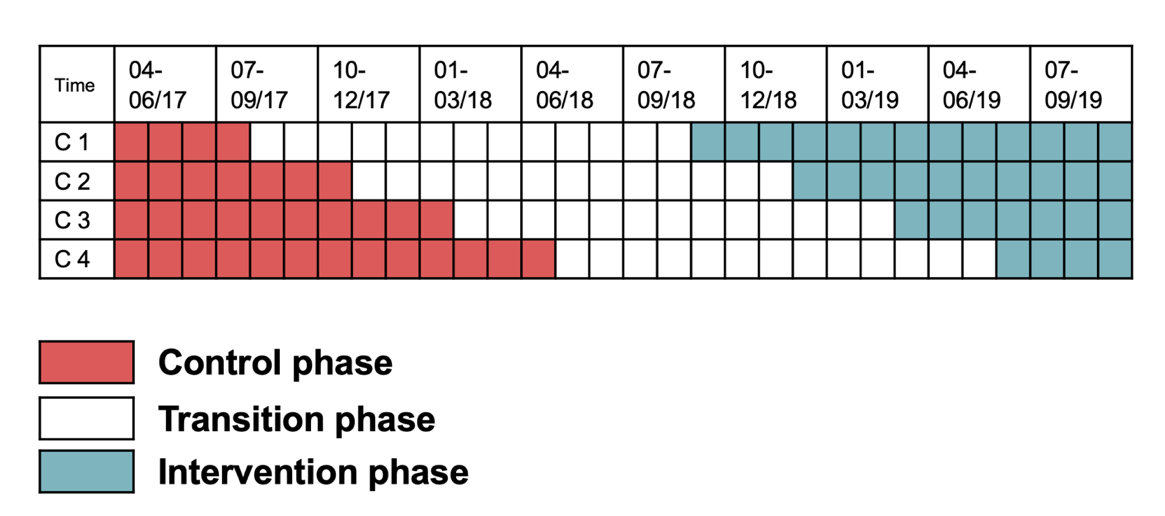

Supplement: Multimedia Appendix 2 [file jmir_v24i3e34098_app2.docx]

Multimedia appendix 5, Study enrolment over time (inpatient sector)


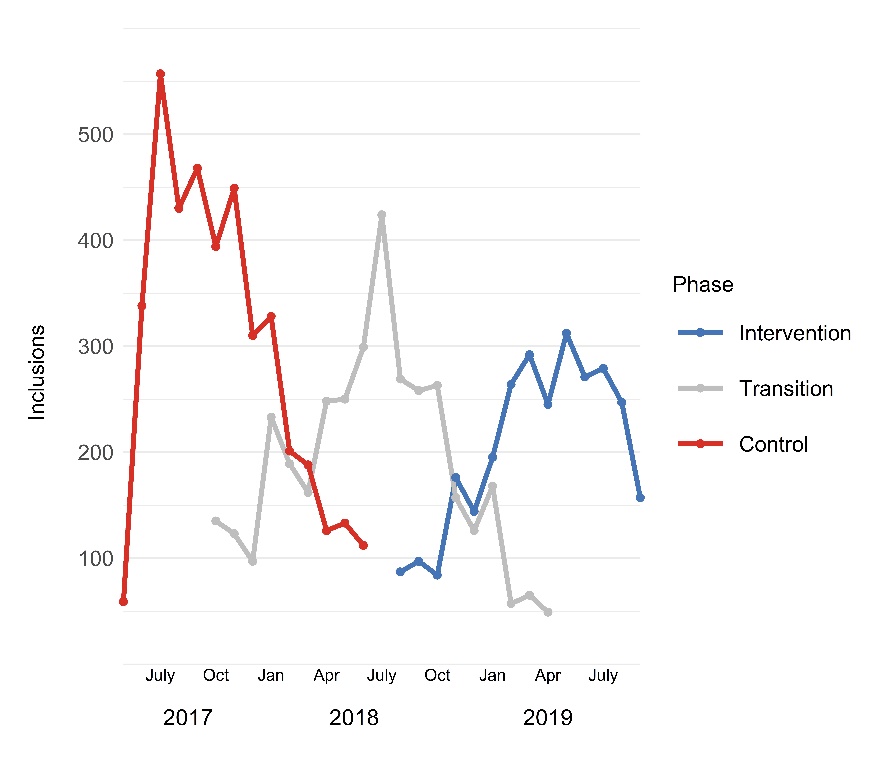

Supplement: Multimedia Appendix 5 [file jmir_v24i3e34098_app5.docx]

Multimedia appendix 9, Study enrolment over time (outpatient sector)


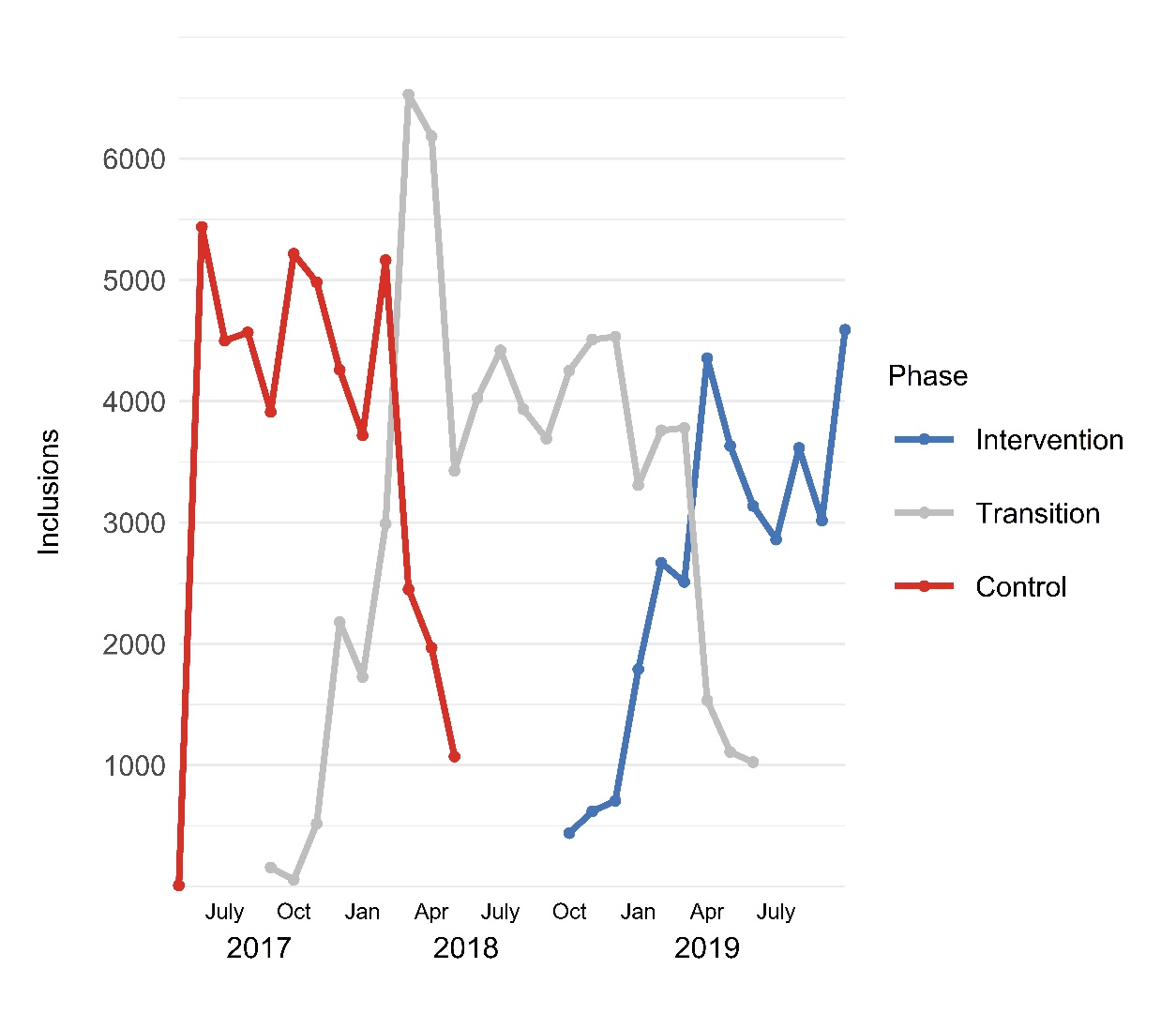

Supplement: Multimedia Appendix 9 [file jmir_v24i3e34098_app9.docx]
