# Supplementary material for: An Innovative Telemedical Network to Improve Infectious Disease Management in Critically Ill Patients and Outpatients (TELnet@NRW): Stepped-Wedge Cluster Randomized Controlled Trial
Source: J Med Internet Res. 2022 Mar 2;24(3):e34098. doi: 10.2196/34098 (PMC8928042; doi:10.2196/34098)
Supplement: Multimedia Appendix 3 [file jmir_v24i3e34098_app3.docx]

Multimedia appendix 3, Analysis algorithms of the 10-Choosing Wisely Recommendations

| Positive recommendations (P1 – P5) | Inpatient sector | Outpatient sector |
| --- | --- | --- |
| *Staphylococcus aureus* bloodstream infection imperatively needs efficacious antimicrobial treatment and identification and elimination of the source of infection. | X |  |
| In critically ill patients with signs of infection, early appropriate antibiotic therapy is crucial after obtaining cultures, and treatment should be regularly re-evaluated. | X |  |
| Annual influenza vaccination should be given to individuals aged >60 years, patients with specific co‐morbidities and people (e.g., health care workers) who may infect vulnerable persons. |  | X |
| All children should receive the measles vaccine, and adults born after 1970 without prior documented vaccination against measles should get at least one dose of the vaccine. |  | X |
| Prescribe oral forms of highly bioavailable antimicrobial agents to patients who can reliably receive and absorb medications via the enteral route. | X |  |
| Negative recommendations (N1 – N5) | | |
| Avoid prescribing antibiotics for uncomplicated upper respiratory tract infections including bronchitis. |  | X |
| Do not treat asymptomatic bacteriuria with antibiotics. | X | X |
| Do not treat Candida recovered from respiratory or gastrointestinal tract specimens. | X |  |
| Do not extend the administration of prophylactic antibiotics after surgery (after the patient has left the operating room). | X |  |
| Do not treat elevated C‐reactive protein (CRP) or procalcitonin levels in serum with antibiotics in patients not presenting signs or symptoms of infection. | X |  |

**Analysis algorithms**

**Inpatient sector**

**P1**

| Pickup: | Staph.-aureus detection by receipt of a positive blood culture for Staph.-aureus (patient is included in the Staph.-aureus data) |
| --- | --- |
| Query: | 1. After enrolment in the study, at least one additional blood culture was taken ( = from Event Number 7 onwards). 2. Identification of the source of infection: at least one imaging procedure (TTE; TEE; X-ray; CT) has occurred after or at study enrolment (=from Event Number 1 but without Event Number 2 to 6). 3. Elimination of the source of infection: Catheter was changed (or surgery entry TEL_M040_SURGERY_Q010) after or during enrolment in the study. 4. Antibiotics: At least 14 days of intravenous antibiotic administration (regardless of substance and, if applicable, change of substance) |
| Population: | All patients included in the Staph.-aureus data |
| Criterion met when: | P1 is considered fulfilled if all points 1-4 are fulfilled.  (Operationalization in Open Clinica: If the patient has at least one entry with the specified/searched value for one of the listed items since the start of the study, then this criterion is considered to be fulfilled). |

**P2**

| Pickup: | Proven septic shock OR severe sepsis (patient has completed Sepsis Bundle) |
| --- | --- |
| Query: | 1. Antibiotic administration after 1 OR 2 OR 3 hours OR preexisting antibiotic therapy in the last 12 hours before diagnosis   Explanation: Criteria of particular infection severity can also occur with a time lag. For example, if pneumonia has been recognized and treated, but gas exchange or the circulatory situation does not deteriorate until the following day, the diagnosis of severe sepsis or septic shock (as a result of the treated pneumonia) will not be made until the above-mentioned disturbances occur.   1. Reevaluation over several days;   Criterion met if a, b, c or d is met:   - 1. more than 1 substance and different starting date (=change has taken place or number has been increased)   2. more than 1 substance and different end date (=change has taken place or number has been reduced)   3. more than 1 substance but not exceeding 4 days max (same start/end date possible)   4. only 1 substance for maximum 4 days   Note: Cases detected in this way have certainly reevaluated based on the data. However, it cannot be detected in the data if someone has reevaluated and concluded that no change needs to be made. This means that there is an unreported number of reevaluated cases that are not recognized as such.  In the televisits, a review of the indication, duration, dosage and adjustment takes place for every patient receiving antibiotic therapy. For the intervention group with teleconsultation, the point reevaluation is therefore always considered to be fulfilled.   1. Blood cultures in hour 0 to 3   Note: The criterion is considered "fulfilled" if a sufficient number (4 bottles) of blood cultures were taken up to three hours after diagnosis. If blood cultures were taken after the start/continuation of antibiotic therapy AND the antibiotic was changed, the criterion is also rated as "fulfilled". This often happens when the severity of the disease increases. This means if the criteria for the particular severity of the infection appeared for the first time during ongoing therapy or if a lack of response to the initial therapy is assumed. |
| Population: | All patients with sepsis bundle completed/applied. |
| Criterion met when: | P2 is considered fulfilled if all points 1-3 are fulfilled. There is also an evaluation of the individual points (1-3).  Note: If a diagnosis is documented on the hour (e.g., 4:00 p.m.) and coincides with the blood culture collection time and antibiotic administration time (also 4:00 p.m.), the correct sequence is assumed. Otherwise, a particularly rapid processing but less accurate documentation would be interpreted as incorrectly low target achievement. |

**P5**

| Pickup: | Administration of the defined targeted antibiotics (ciprofloxacin, clindamycin, levofloxacin, trimethoprim-  sulfametoxazole) |
| --- | --- |
| Query: | 1. Exclusion of patients with contraindication: meningitis, staph. aureus bacteremia, endocarditis, sepsis, swallowing disorders   Note: Without a temporal connection to the antibiotic administration, i.e. if one of the above diagnoses was documented at any time, the person is excluded from the assessment of P5   1. Intravenous or oral administration of the antibiotic (to be merged via event number) |
| Population: | Patients who have been administered the antibiotics listed in point 1 and who do not have a contraindication |
| Criterion met when: | P5 is considered fulfilled if the antibiotic administration ( point 2) was oral and not intravenous.  Note: Change of route of administration (i.e., from oral to lead compound IV or from IV to oral) are also considered "met." |

**N2**

| Pickup: | Patients with a documented receipt of a positive urine specimen |
| --- | --- |
| Query: | 1. Exclusion of patients according to SIRS criteria for temperature and/or leukocytes (24 h +/- receipt of findings, pooling of the values via the event number):    1. Temperature: exclusion if the body temperature within 24 hours before and after receipt of the results of the urine sample (with the finding "evidence of bacteria in the urine") was above 38 or below 36 degrees, which means exclusion if the following applies: lowest body temperature of the day is above or equal to 38 degrees or the highest temperature of the day is below or equal to 36 degrees    2. Leukocytes: exclusion if the leukocyte count in the blood within 24 hours before and after receipt of the results was above 12000 or below 4000 2. Exclusion of patients who had a SOFA score > 3 on the day of receipt of the findings. 3. If an antibiotic was given with the statement "Bacteria in urine", "No explanation documented", or "Reason not known", or is the indication of a reason completely missing? |
| Population: | Patients with a positive urine result for whom the SIRS criteria for temperature and/or leukocytes were not met 24 hours before and after the receipt of the result and who did not have a SOFA score > 3 on the day of the finding |
| Criterion met when: | N2 is considered to be met if point 3 can be answered with no (the criterion is considered to be met even if the patient receives antibiotics but the reason for this is different from those mentioned in point 3). |

**N3**

| Pickup: | Patients with a Candida detection in the feces or respiratory specimen |
| --- | --- |
| Query: | 1. Exclusion of patients who also have Candida detection in another specimen (blood, tissue, punctate/secretions, cerebrospinal fluid) (without temporal relationship to the fecal specimen/ respiratory material specimen) 2. If the patient was given an antimycotic drug with the justification "Candida in the respiratory tract", "Candida in the feces", "No reason documented" or "Reason not known", or is the indication of a rationale completely missing? (without temporal connection to the fecal specimen/specimen of the respiratory material) |
| Population: | Patients with Candida detection in feces or respiratory specimen without further Candida detection |
| Criterion met when: | N3 is considered to be fulfilled if point 2 can be answered with no. |

**N4**

| Pickup: | Patients with surgery |
| --- | --- |
| Query: | 1. Exclusion of patients with    1. a documented infection (infection in CNS, endocaritis, meningitis, sepsis, cardiac, vascular, circulatory, gastro, pulmonary, urinary tract, skin) or a suspected infection or intraoperative specimen, if it was present at the same time as the day of antibiotic administration (merging of the information about the event number) or    2. a SOFA score > 3 on the antibiotic day (merging of the information on the event number) 2. If a post-op antibiotic was given with the rationale "Perioperative", "No reason documented" or "Reason not known" or is the indication of a reason completely missing? |
| Population: | Patients with surgery but without infection, suspected infection, intraoperative specimens or SOFA score > 3 on the day of antibiotic administration |
| Criterion met when: | N4 is considered to be fulfilled if point 2 can be answered with no. |

**N5**

| Pickup: | Patients with elevated CRP (CRP >= 5) |
| --- | --- |
| Query: | 1. Exclusion of patients with    1. infections (no consideration of the temporal relationship to the increased CRP)    2. SOFA score > 3 (temporal relationship to the day of antibiotic administration and elevated CRP above Event Number) 2. If a postoperative antibiotic was used with the justification "Elevated CRP and/or PCT", "No reason documented" or "Reason not known" or is the indication of a reason completely missing? |
| Population: | Patients with elevated CRP but no diagnosed infection or SOFA score > 3 on the day of antibiotic administration.  Note: Patients with elevated PCT were not included because, according to the consortium leadership, determination of PCT in basic and standard care hospitals is infrequent. |
| Criterion met when: | N5 is considered to be fulfilled if point 2 can be answered with no. |

**Outpatient sector**

**P3 and P4**

To capture any effects of the telemedicine intervention on the vaccination behavior of the participating outpatient physicians, quarterly, physician-related influenza and measles vaccination rates were analyzed on the basis of additionally obtained billing data from the Kassenärztliche Vereinigungen (KV) Nordrhein and Westfalen-Lippe.

**N1**

| Determination of the analysis sample: | Study sample: patients with an infection in the upper respiratory tract. |
| --- | --- |
| Determination of patients with upper respiratory tract infection: | All patients are included in the analysis for whom os (organ system) = 8 (upper respiratory tract)  Note: Only the respiratory tract can be mapped as an affected organ system. Information on whether the infection is "uncomplicated" cannot be obtained from the data. |
| Determine whether N1 is fulfilled: | Antibiotic administration: v_ab = Antibiotic administration: 0 = no, 1 = yes  If v_ab = 1, N1_fuflilled = 0,  If y_ab = 0, N1_fulfilled = 1,  Otherwise NA |
| Method of analysis: | Evaluation using a logistic regression model with estimation of a learning effect curve from teleconsultations under control for individual physicians. |

**N2**

| Determination of the analysis sample: | Study sample: patients with an asymptomatic bacteriuria. |
| --- | --- |
| Determination of patients with asymptomatic bacteriuria: | All patients are included in the analysis for whom abc (asymptomatic bacteriuria) = 1 (present) |
| Determine whether N2 is fulfilled: | Antibiotic administration: v_ab = Antibiotic administration: 0 = no, 1 = yes  If v_ab = 1, N2_fuflilled = 0,  If y_ab = 0, N2_fulfilled = 1,  Otherwise NA |
| Method of analysis: | Evaluation using a logistic regression model with estimation of a learning effect curve from teleconsultations under control for individual physicians. |
