# Supplementary material for: An Innovative Telemedical Network to Improve Infectious Disease Management in Critically Ill Patients and Outpatients (TELnet@NRW): Stepped-Wedge Cluster Randomized Controlled Trial
Source: J Med Internet Res. 2022 Mar 2;24(3):e34098. doi: 10.2196/34098 (PMC8928042; doi:10.2196/34098)
Supplement: Multimedia Appendix 4 [file jmir_v24i3e34098_app4.docx]

Multimedia appendix 4, Model structures

Model structure primary data for inpatient care

$$\boldsymbol{ln}\frac{\boldsymbol{P(}\boldsymbol{Y}_{\boldsymbol{p}}\boldsymbol{=1)}}{\boldsymbol{P}\left( \boldsymbol{Y}_{\boldsymbol{p}}\boldsymbol{=0} \right)}\boldsymbol{=}\boldsymbol{\beta}_{\boldsymbol{1}\boldsymbol{i}}\boldsymbol{*}\boldsymbol{KH}_{\boldsymbol{ip}}\boldsymbol{+}\boldsymbol{\beta}_{\boldsymbol{2}}\boldsymbol{*}\boldsymbol{Age}_{\boldsymbol{p}}\boldsymbol{+}\boldsymbol{\beta}_{\boldsymbol{3}}\boldsymbol{*}\boldsymbol{SOFA}_{\boldsymbol{p}}\boldsymbol{+}\boldsymbol{\beta}_{\boldsymbol{4}\boldsymbol{j}}\boldsymbol{*}\boldsymbol{Group}_{\boldsymbol{jp}}\boldsymbol{+}\boldsymbol{\varepsilon}_{\boldsymbol{p}}$$

i = Count variable hospital, p = Count variable patient, j = Count variable group (IG with/without teleconsultation)

Model structure primary data for outpatient care

$$\boldsymbol{ln}\frac{\boldsymbol{P(}\boldsymbol{Y}_{\boldsymbol{p}}\boldsymbol{=1)}}{\boldsymbol{P}\left( \boldsymbol{Y}_{\boldsymbol{p}}\boldsymbol{=0} \right)}\boldsymbol{=}\boldsymbol{\beta}_{\boldsymbol{1}\boldsymbol{i}}\boldsymbol{*}\boldsymbol{Physician}_{\boldsymbol{ip}}\boldsymbol{+}\boldsymbol{\beta}_{\boldsymbol{2}}\boldsymbol{*}\boldsymbol{Age}_{\boldsymbol{p}}\boldsymbol{+}\boldsymbol{\beta}_{\boldsymbol{3}}\boldsymbol{*}\boldsymbol{Group}_{\boldsymbol{p}}\boldsymbol{+}\boldsymbol{\beta}_{\boldsymbol{4}}\boldsymbol{*}\boldsymbol{n}_{\boldsymbol{p}}\boldsymbol{+}\boldsymbol{\beta}_{\boldsymbol{5}}\boldsymbol{*}{\boldsymbol{n}_{\boldsymbol{p}}}^{\boldsymbol{2}}\boldsymbol{+}\boldsymbol{\varepsilon}_{\boldsymbol{p}}$$

i = Count variable outpatient physician, p = Count variable patient

Model structure secondary data for outpatient care

$$\boldsymbol{ln}\left( \boldsymbol{vaccination rate}_{\boldsymbol{ilk}} \right)\boldsymbol{=}\boldsymbol{\beta}_{\boldsymbol{1}\boldsymbol{i}}\boldsymbol{*}\boldsymbol{Physician}_{\boldsymbol{i}}\boldsymbol{+}\boldsymbol{\beta}_{\boldsymbol{2}}\boldsymbol{*}\boldsymbol{Group}_{\boldsymbol{ilk}}\boldsymbol{+}\boldsymbol{\beta}_{\boldsymbol{3}}\boldsymbol{*}\boldsymbol{Cases}_{\boldsymbol{ilk}}\boldsymbol{+}\boldsymbol{\beta}_{\boldsymbol{4}\boldsymbol{l}}\boldsymbol{*}\boldsymbol{Quarter}_{\boldsymbol{il}}\boldsymbol{+}\boldsymbol{\varepsilon}_{\boldsymbol{ilk}}$$

i = Count variable outpatient physician, l = Count variable quarter, k = count variable year
