# Supplementary material for: An Innovative Telemedical Network to Improve Infectious Disease Management in Critically Ill Patients and Outpatients (TELnet@NRW): Stepped-Wedge Cluster Randomized Controlled Trial
Source: J Med Internet Res. 2022 Mar 2;24(3):e34098. doi: 10.2196/34098 (PMC8928042; doi:10.2196/34098)
Supplement: Multimedia Appendix 6 [file jmir_v24i3e34098_app6.docx]

Multimedia appendix 6, Missing value distribution of baseline SOFA scores

|  | Missing values before imputation | Missing values after imputation |
| --- | --- | --- |
| Respiratory system | 1,143 (11^.^09%) | 795 (7^.^71%) |
| Coagulation | 1,202 (11^.^66%) | 418 (4^.^06%) |
| Liver | 4,643 (45^.^06%) | 3,148 (30^.^55%) |
| Cardiovascular system | 70 (0^.^68%) | 50 (0^.^49%) |
| Nervous system | 2,95 (2^.^86%) | 225 (2^.^18%) |
| Kidneys | 1,593 (15^.^56%) | 548 (5^.^32%) |
| Total (baseline SOFA scores with at least one sub-score missing) | 5,274 (51^.^18%) | 3,681 (35^.^72%) |
