# Supplementary material for: An Innovative Telemedical Network to Improve Infectious Disease Management in Critically Ill Patients and Outpatients (TELnet@NRW): Stepped-Wedge Cluster Randomized Controlled Trial
Source: J Med Internet Res. 2022 Mar 2;24(3):e34098. doi: 10.2196/34098 (PMC8928042; doi:10.2196/34098)
Supplement: Multimedia Appendix 7 [file jmir_v24i3e34098_app7.docx]

Multimedia appendix 7, Regression analyses of sepsis bundle compliance

| **Sepsis bundle** | **0-6 hours (N=227)** | | | **0-3 hours (N=227)^a^** | | | **4-6 hours (N=190)**^b^ | | |
| --- | --- | --- | --- | --- | --- | --- | --- | --- | --- |
|  | Compliance n/N (%) | OR (95% CI) | p value | Compliance n/N (%) | OR (95% CI) | p value | Compliance n/N (%) | OR (95% CI) | p value |
| **Control variables** |  |  |  |  |  |  |  |  |  |
| SOFA score | - | 0.958 | .36 | - | 1.003 | .96 | - | 0.967 | .59 |
|  |  | (0.874, 1.050) |  |  | (0.913, 1.102) |  |  | (0.856, 1.094) |  |
| Age | - | 0.996 | .77 | - | 0.992 | .56 | - | 1.017 | .32 |
|  |  | (0.972, 1.021) |  |  | (0.965, 1.019) |  |  | (0.984, 1.052) |  |
| **Group variables** |  |  |  |  |  |  |  |  |  |
| Control group | 17/66 | Ref | - | 35/66 | Ref | - | 20/57 | Ref | - |
|  | (25.8%) |  |  | (53.0%) |  |  | (35.1%) |  |  |
| Intervention group |  |  |  |  |  |  |  |  |  |
| without teleconsultation | 84/138 | 4.315 | <.001 | 98/138 | 2.174 | .04 | 90/115 | 8.289 | <.001 |
|  | (60.9%) | (2.083, 9.308) |  | (71.0%) | (1.052, 4.551) |  | (78.3%) | (3.499, 21.176) |  |
| with teleconsultation | 17/23 | 7.739 | .001 | 18/23 | 3.326 | .07 | 15/18 | 14.245 | .001 |
|  | (73.9%) | (2.379, 28.026) |  | (78.3%) | (0.973, 12.919) |  | (83.3%) | (3.121, 85.424) |  |
| - not applicable; *CI* confidence interval; *OR* odds ratio; *Ref* reference group; Each model also controlled for hospital-specific effects, which are not reported individually in this table; CIs were calculated based on profile likelihood estimation; ^a^ Items in the 3-hour bundle include: measurement of lactate levels, blood cultures obtained prior to administration of antibiotics, administration of broad-spectrum antibiotics, and administration of 30 mL/kg crystalloid fluid for hypertension or lactate levels ≥4 mmol/L; ^b^ Items in the 6-hour bundle include: application of vasopressors for persistent hypotension (to maintain a mean arterial pressure ≥65 mmHg), assessment of central venous pressure and central venous oxygen saturation, and remeasurement of lactate levels if initially elevated | | | | | | | | | |
