# Supplementary material for: An Innovative Telemedical Network to Improve Infectious Disease Management in Critically Ill Patients and Outpatients (TELnet@NRW): Stepped-Wedge Cluster Randomized Controlled Trial
Source: J Med Internet Res. 2022 Mar 2;24(3):e34098. doi: 10.2196/34098 (PMC8928042; doi:10.2196/34098)
Supplement: Multimedia Appendix 8 [file jmir_v24i3e34098_app8.docx]

Multimedia appendix 8, Regression analysis of acute respiratory distress syndrome therapy compliance

|  | **ARDS mild (N = 427) ^a^** | | | **ARDS moderate (N=423) ^a^** | | | **ARDS severe (N =132) ^a^** | | |
| --- | --- | --- | --- | --- | --- | --- | --- | --- | --- |
|  | Compliance n/N (%) | OR (95%-CI) | p value | Compliance n/N (%) | OR (95%-KI) | p value | Compliance n/N (%) | OR (95%-CI) | p value |
| **Control variables** |  |  |  |  |  |  |  |  |  |
| SOFA-Score | - | 0.987 | .82 | - | 0.975 | .63 | - | 0.936 | .46 |
|  |  | (0.884; 1,100) |  |  | (0.876; 1.081) |  |  | (0.79; 1.114) |  |
| Age | - | 0.973 | .04 | - | 0.985 | .21 | - | 0.959 | .09 |
|  |  | (0.949; 0.998) |  |  | (0.962; 1.009) |  |  | (0.912; 1.006) |  |
| **Group variables** |  |  |  |  |  |  |  |  |  |
| Control group | 16/217 | Ref |  | 26/219 | Ref |  | 10/61 | Ref |  |
|  | (7.4%) |  |  | (11.9%) |  |  | (16.4%) |  |  |
| Intervention group |  |  |  |  |  |  |  |  |  |
| without teleconsultation | 9/49 | 3.621 | .02 | 9/46 | 1.949 | .17 | 4/14 | 1.169 | .86 |
|  | (18.4%) | (1.256; 10.319) |  | (19.6%) | (0.723; 4.995) |  | (28.6%) | (0.181; 6.714) |  |
| with teleconsultation | 19/161 | 2.355 | .04 | 20/158 | 1.214 | .59 | 8/57 | 0.555 | .39 |
|  | (11.8%) | (1.023; 5.516) |  | (12.7%) | (0.595; 2.462) |  | (14.0%) | (0.139; 2.070) |  |
| - not applicable; *CI* confidence interval; *OR* odds ratio; *Ref* reference group.  **^a^** Each model also controlled for hospital-specific effects, which are not reported individually in this table; CIs were calculated based on profile likelihood estimation. | | | | | | | | | |
