# Supplementary material for: An Innovative Telemedical Network to Improve Infectious Disease Management in Critically Ill Patients and Outpatients (TELnet@NRW): Stepped-Wedge Cluster Randomized Controlled Trial
Source: J Med Internet Res. 2022 Mar 2;24(3):e34098. doi: 10.2196/34098 (PMC8928042; doi:10.2196/34098)
Supplement: Multimedia Appendix 10 [file jmir_v24i3e34098_app10.docx]

Multimedia appendix 10, Regression analyses of secular effects on inpatient primary outcomes

|  | **P1^a^ (N=298)** | | **P2^b^ (N=476)** | | **P5^c^ (N=188)** | | **N4^d^ (N=319)** | | **N5^e^ (N=1,307)** | |
| --- | --- | --- | --- | --- | --- | --- | --- | --- | --- | --- |
|  | OR (95% CI) | P value | OR (95% CI) | P value | OR (95% CI) | P value | OR (95% CI) | p value | OR (95% CI) | P value |
| **Control variables** |  |  |  |  |  |  |  |  |  |  |
| SOFA score |  |  | 0.9746 | .52 | 1.2686 | .01 | 0.9442 | .66 | 0.7837 | .01 |
|  |  |  | (0.9016, 1.0534) |  | (1.0600, 1.5386) |  | (0.7377, 1.2389) |  | (0.6500, 0.9430) |  |
| Age | 0.9898 | .31 | 0.9836 | .13 | 0.9853 | .36 | 1.0344 | .01 | 0.9884 | .07 |
|  | (0.9701, 1.0097) |  | (0.9623, 1.0044) |  | (0.9547, 1.0179) |  | (1.0070, 1.0636) |  | (0.9758, 1.0007) |  |
| **Time variables** |  |  |  |  |  |  |  |  |  |  |
| Days since start of the study | 1.0095 | .003 | 0.9955 | .03 | 0.9974 | .28 | 0.9968 | .16 | 1.0000 | .98 |
|  | (1.0036, 1.0165) |  | (0.9913, 0.9995) |  | (0.9926, 1.0021) |  | (0.9920, 1.0010) |  | (0.9975, 1.0025) |  |
| Days since start of the transition phase | 0.9914 | .02 | 1.0099 | <.001 | 1.0018 | .59 | 1.0060 | .09 | 1.0012 | .45 |
|  | (0.9835, 0.9982) |  | (1.0046, 0.0154) |  | (0.9952, 1.0086) |  | (0.9993, 1.0132) |  | (0.9981, 1.0045) |  |
| *CI* confidence interval; OR odds ratio; Each model also controlled for hospital specific effects, which are not reported individually in this table; CIs were calculated based on profile likelihood estimation.  Primary Outcomes:  ^a^ P1 Imperatively start antimicrobial treatment and remove the focus on Staphylococcus aureus bloodstream infection,  ^b^ P2 Critically ill patients with signs of infection need early appropriate antibiotic therapy,  ^c^ P5 Prefer oral formulations of highly bioavailable antimicrobials whenever possible,  ^d^ N4 Do not prolong prophylactic administration of antibiotics in patients after they have left the operating room,  ^e^ N5 Do not treat an elevated C-reactive protein (CRP) or procalcitonin level with antibiotics in patients without signs of infection. | | | | | | | | | | |
