# Supplementary material for: An Innovative Telemedical Network to Improve Infectious Disease Management in Critically Ill Patients and Outpatients (TELnet@NRW): Stepped-Wedge Cluster Randomized Controlled Trial
Source: J Med Internet Res. 2022 Mar 2;24(3):e34098. doi: 10.2196/34098 (PMC8928042; doi:10.2196/34098)
Supplement: Multimedia Appendix 11 [file jmir_v24i3e34098_app11.docx]

**Multimedia Appendix 11 – TELnet@NRW Study Group.**

**University Hospitals**

**Aachen** (Department of Operative Intensive Medicine and Intermediate Care, University Hospital Aachen, Aachen, Germany): Gernot Marx, Robert Deisz, Miriam Haverkamp, Fabian Juzek-Küpper, Susanne Rademacher, Jennifer Kistermann, Nadja Binz, Eva Steinfeld, Jan Wienströer, Yvonne Schumacher, Alexander Schnitzler, Sandra Dohmen, Holger Wilms, Anke Offermann, Sven Bellen, Ina Huppertz, Marc Kaiser, Judith Ibba, Evangelos Karasimos, Henna Schulze-Steinen, Joachim Köck, Julia Amkreutz, Volker Lowitsch, Arndt Czepluch, Waldemar Ostrowski; (Department of Infectious Diseases and Infection Control, University Hospital Aachen, Aachen, German): Sebastian Lemmen, Miriam Haverkamp, Fabian Juzek-Kuepper

**Münster** (Department of Anaesthesiology, Intensive Care Medicine and Pain Medicine, University Hospital Münster, Münster, Germany): Kathrin Sperling, Jan Sönke Englbrecht, Daniela Bause, Kai Börner, Niko Brenscheid, Ursula Maria Volkenhoff, Hanna Maria Mittag, Juliane Hecke, Antje Gottschalk; (Office for eHealth): Christian Juhra, Andrea Kuckuck-Winkelmann, Martin Schnitt; (Institute for Hygiene/Antibiotic Stewardship): Christian Lanckohr, Marcus Ahrends, Dagmar Horn.

**Participating hospitals**

**Aachen** (Department of Anaesthesiology, Franziskus-Hospital, University Hospital Aachen, Aachen, Germany): Barbara Sauerzapfe, Barbara Stolzenberg.

**Arnsberg** (Department of Anaesthesiology and Intensive Care, Karolinen-Hospital & Marien-Hospital, Hospital Hochsauerland GmbH, Arnsberg, Germany): Timur Sellmann, Martin Heinrich Christian Bredendiek, Michael Steinberg, Dörte Van der Smissen, Martin Sander, Robert Pannewitz, Christina Nagel, Astrid Hönninger.

**Coesfeld** (Department of Anaesthesiology, Christophorus Kliniken Coesfeld, Coesfeld, Germany): Martin Mühlmeyer, Irene Tzanova, Jan Hakenbeck, Lutz von Müller (Institute of Laboratory Medicine, Microbiology and Hygiene), Christian Deiters.

**Dormagen** (Department of Anaesthesiology, Intensive Medicine & Emergency Medicine, Rheinland Klinikum Dormagen, Dormagen, Germany): Britta Jansen, Stefan Soltész.

**Düren** (Department of Anaesthesiology and Intensive Care, Hospital Düren gem. GmbH, Düren, Germany): Stefan Schröder, Olaf Schröder, Jan Karl Schütte, Bernhard Heising (Centre for Infectiology and Hospital Hygiene).

**Geilenkirchen** (Department of Anaesthesiology and Intensive Care, St. Elisabeth Hospital Geilenkirchen, Geilenkirchen, Germany): Sven Reckort, Stefan Pinger (Department of Internal Medicine), Christof Fleischer, Georg Gunesch.

**Grevenbroich** (Department of Anaesthesiology, Intensive Medicine & Emergency Medicine, Elisabeth Hospital Grevenbroich, Rheinland Klinikum, Grevenbroich, Germany): Klaus Benner, Ulrike Gründel, Roland Lorenz.

**Gronau** (Department of Anaesthesiology, Operative Intensive Medicine and Pain Therapy, St. Antonius-Hospital Gronau GmbH, Gronau, Germany): Thomas Hugo Gurk, Tin Tucman, Marina Hemming, Petra Stenau.

**Jülich** (Department of Anaesthesiology, St. Elisabeth Hospital Jülich, Jülich, Germany): Rudolf Jegen, Marcus Flucht, Christian Dreyer.

**Linnich** (Department of Anaesthesiology, St. Josef-Krankenhaus Linnich, Linnich, Germany): Sabine Tack, Volker Braun, Birgit Nose-Dreßen.

**Münster** (Department of Anaesthesiology, Intensive Care Medicine and Pain Medicine, Evangelisches Krankenhaus Johannisstift Münster gGmbH, Münster, Germany): Verena Lange, Marco Maria Ralf Max Franciscus Püschel, Uwe Hellwig, Christian Hadem.

**Rheine** (Department of Anaesthesiology and Intensive Care, Jakobi-Hospital Rheine, Rheine, Germany): Angela Margarete Grote-Reith, Niels Wolter, Michelle Smyrek, Thomas Meemann.

**Simmerath** (Department of Anaesthesiology and Intensive Care, Eifelklinik St. Brigida, Simmerath, Germany): Muhamad Badreddin, Rudolf Stollenwerk, Esamddin Alarabi, Kerstin Schwark.

**Steinfurt** (Department of Anaesthesiology and Intensive Care Medicine, UKM Marienhospital Steinfurt GmbH, Steinfurt, Germany): Ulrich Ruta, Wolfram Kraft, Christina Smok, Holger Jochen Albert Janssen.

**Stolberg** (Department of Internal Medicine, Bethlehem Gesundheitszentrum Stolberg gGmbH, Stolberg, Germany): Andreas Bootsveld, Wolfgang Bilke (Department of Anaesthesiology), Bernd Meuthen, Christoph Dietrich, Werner Krumholz (Department of Anaesthesiology).

**Warendorf** (Department of Anaesthesiology, Intensive Care and Pain Medicine, Josephs-Hospital Warendorf, Warendorf, Germany): Alexander Reich, Tobias Mock, Heike Dreimann, Thomas Philipp Weber.

**Physician networks**

**MuM Medizin und Mehr eG**: Hans Jürgen Beckmann, Bettine Beinert, Heinz-Georg Beneke, Torsten Bode, Martin Bünemann, Ralf Camnitius, Reinhard Dolle, Dieter Döpke, Michael Droste, Gesa Fiedler, Nadine Fischer, Stefan Gerdau, Hanns Grübbel, Simone Grünzig, Daniel Gundlach, Thomas Herzig, Eckhard Jungmann, Nicole Kammann, Henrik Könemann, Gabriele Könemann, Kai Könemann, Kerstin Köplin-Fritsche, Wiebke Krüger, Heike Krüger, Cesary Krüger, Achim Küffmeier, Christian Lücker, Elke Lützow, Ernst-Friedrich Maschmann, Marcus Nippesen, Cordula Rautenberg, Thomas Rittmann, Peter Rosellen, Andreas Schimke, Ralf Twele, Detlef Walter, Sabine Walter, Bruno Weil, Rolf Wilmsmeier, Sebastian Ziegler, Detlef Ziemann, Wilhelm Fischer, Bernd Roß, Annette Hempen, Elena Kruse.

**Gesundheitsnetz Köln-Süd (GKS) e.V**.: Dietmar Ederer-Merdian, Manuela Klinkhamels, Georg Schmitz, Désirée Dahmen, Michael König, Roger Schmid, Kirsten Benn, Ulla Woschée, Miriam Koeller-Bratz, Rodica Dornaika, Stephan Schmitz, Kai Severin, Tillmann Steinmetz, Ulf Esser, Stefan Keymis, Peter Sedlaczek, Christian Flügel-Bleienheuft, Hubertus Meyer-Madaus, Klaus Schott, Friedrich Worth, Stefan Christmann, Isabel Eßer, Heinz-Günther Göddertz, Stephan Leuwer, Gero Quante, Sandro Meider, Ralf Krott, Frauke Sulimma, Norbert Schnitzler, Julika Hiersemann, Thomas Schramm, Martin Warlich, Maria Jesse, André Seeliger, Stephan Carl Wenzel, Bettina Squarra-Rohde, Christine Emmerich, Paul Mackert, Bernhard Schiffgen, Sebastian Effinger, Philipp Richrath, Margret Nawroth, Karin Wolf, Hilke Betten, Patrick Hamm, Rudolf Stratmeyer, Ansgar Maroldt, Silke Riebensahm, Nicola Pega-Wolter, Christian Windelen, Claudia Rodermann.
